# Fast and Simple Analytical Method for Direct Determination of Total Chlorine Content in Polyglycerol by ICP-MS

**DOI:** 10.3390/molecules23020487

**Published:** 2018-02-23

**Authors:** Agata Jakóbik-Kolon, Andrzej Milewski, Piotr Dydo, Magdalena Witczak, Joanna Bok-Badura

**Affiliations:** 1 Faculty of Chemistry, Silesian University of Technology, Krzywoustego 6, 44-100 Gliwice, Poland; andrzej.milewski@polsl.pl (A.M.); piotr.dydo@polsl.pl (P.D.); joanna.bok-badura@polsl.pl (J.B.-B.); 2 Wood Technology Institute, Bioenergy Department, Winiarska 1, 60-654 Poznan, Poland; m_witczak@itd.poznan.pl

**Keywords:** chlorine, chloride, polyglycerol, inductively coupled plasma mass spectrometry, simple sample preparation

## Abstract

The fast and simple method for total chlorine determination in polyglycerols using low resolution inductively coupled plasma mass spectrometry (ICP-MS) without the need for additional equipment and time-consuming sample decomposition was evaluated. Linear calibration curve for ^35^Cl isotope in the concentration range 20–800 µg/L was observed. Limits of detection and quantification equaled to 15 µg/L and 44 µg/L, respectively. This corresponds to possibility of detection 3 µg/g and determination 9 µg/g of chlorine in polyglycerol using studied conditions (0.5% matrix-polyglycerol samples diluted or dissolved with water to an overall concentration of 0.5%). Matrix effects as well as the effect of chlorine origin have been evaluated. The presence of 0.5% (m/m) of matrix species similar to polyglycerol (polyethylene glycol—PEG) did not influence the chlorine determination for PEGs with average molecular weights (MW) up to 2000 Da. Good precision and accuracy of the chlorine content determination was achieved regardless on its origin (inorganic/organic). High analyte recovery level and low relative standard deviation values were observed for real polyglycerol samples spiked with chloride. Additionally, the Combustion Ion Chromatography System was used as a reference method. The results confirmed high accuracy and precision of the tested method.

## 1. Introduction

Oligoglycerols are commonly used in the food, pharmaceutical, and cosmetic industries. They are mainly used as emulsion stabilizers and dispersants to improve smoothness and provide lubrication. Furthermore, polyglycerol-based products are used in the form of their esters derivatives, which are obtained in reaction with fatty acids [1,2,3]. These esters are utilized mainly in foodstuffs industry as fat-substitutes, emulsifiers, and active agents for the maintenance of foodstuffs’ rheology. Commercially available oligoglycerols are provided mainly by Solvay Chemicals Corporation as diglicerol, polyglycerol-3, or polyglycerol-4 and are composed of a mixture of oligomers in which major components are polyglycerol (PGL) isomers consisting of two, three, or four glycerol units combined, respectively. Thus commercially available oligoglycerols are often the mixtures of various isomers [1,3].

Usually, for industry-scale production, the oligoglycerols are obtained by the epichlorohydrin route [3]. Epichlorohydrin is hydrolyzed to glycidol by sodium hydroxide in aqueous solution, which results in product contamination with chloride salt. Simultaneously, glycerol is formed and reacts with glycidol resulting in diglycerols. Alternately, chlorine containing diglycerol derivate may be produced by the reaction of glycerol with epichlorohydrin. Therefore, the main disadvantage of epichlorohydrin route is that the product contains chlorocarbon compounds and chloride salts, which are undesirable and limited in the final product. Since there are no specific regulations regarding chlorine content in polyglycerols applied in the food and cosmetic industries, one may follow the EU (European Council) laying down specification for glycerol as a food additive, which limits total the chlorine content at 30 µg/g level [4].

The halocarbon derivatives are potentially toxic for the environment and human health. Therefore, purification steps are necessary in order to decrease the chlorine content in the polymeric materials. To control the efficiency of purification processes and monitor the quality of the product, a fast, simple, and reliable method for chlorine determination in polyglycerol and similar oligoetherolic products is required.

Analysis of halogens in organic matrices usually are conducted by decomposition of the samples or salt extraction and further analysis as inorganic ions (e.g., chlorides) by ion chromatography (IC) [5,6,7,8], potentiometric titration [9,10], and inductively coupled plasma mass spectrometry (ICP-MS) apparatus with special equipment [5,6,10,11,12,13,14,15]. Traditional acid digestion is undesirable for this purpose due to possibility of analyte losses by volatilization and chlorine liberation. The evolved chlorine has to be absorbed in alkaline solution or solid (e.g., sodium carbonate) [7,8,16,17,18,19,20]. In such a case, special equipment is used for sample decomposition without volatile analyte loss—Schoniger combustion flask, combustion tubes, microwave-induced combustion system (MIC) [7,8,18,19], or bombs (Parr bomb) [5]. Fusion with sodium carbonate or pyrohydrolysis may also be applied [5,6]. Decomposition of the sample may also be conducted by applying halogen-dedicated ovens for IC or ETV (electrothermal vaporization) adapters for high resolution ICP-MS spectrometers [15,21]. All sample decomposition methods, as additional steps in analysis, increase the time and costs of analysis (especially purchase of additional equipment and reagents of high purity) and constitutes a likely source of various determination errors (e.g., by sample contamination or analyte losses). Rarely are samples just dissolved in water or mixture of solvents and introduced directly into the plasma of ICP-MS [11,20]. Determination of chlorine by this technique is believed to be limited by spectral interferences—all the stable isotopes of chlorine, 35 and 37, are overlapped by plasma-derived species—^18^O^16^O^1^H and ^1^H^36^Ar, respectively [11]. Therefore, an apparatus with high resolution detector (HR ICP-MS) [11,12] or additional equipment (collision cell or dynamic reaction cell) [5] is used to make these interferences negligible. Although new ICP-MS spectrometers rather fulfill these requirements, there are still a lot of working apparatuses—e.g., from before 10 years—which have no such equipment. Additionally, non-spectral interferences in the sample introduction system and in plasma may be elicited by the presence of matrix. Matrix may affect analyzed solution density and viscosity, thus affecting its transport rate as well as analyte ionizability. Non-spectral interferences are also elicited in sample introduction system (memory effects, adsorption, deposition of solids on the cones) as well as in the plasma. Matrix constituents may have an effect on the properties of the plasma and, as a result, shifts in the ion/atom equilibrium may be observed. Generally, such interferences cause suppression of the signal, but sometimes they can cause also signal amplification [22]. This is the so-called “matrix effect” and may be overcome by decomposition of the sample with the above-mentioned methods, by using internal standard, by preparing standards with matrix of the same composition as of samples or by standard addition procedure for calibration [22]. All of them need additional, high purity, and costly reagents or significantly increase analysis time.

There are no reports on determination of chlorine in polyglycerols or similar organic compounds at µg/g level by direct ICP-MS method using a simple, low resolution mass-spectrometer. Therefore, in our studies, the method of chlorine in polyglycerol determination has been evaluated, employing a commonly used ICP-MS spectrometer with low-resolution detector that does not require any additional equipment or time-consuming sample decomposition procedures. This objective was achieved by identifying and minimizing the influence of spectral and non-spectral interferences on the analysis results. This allowed us to elaborate a cheap, fast, simple and reliable analytical method for chlorine determination in polyglycerols. Simple, fast, and cost-effective analytical methods which concern trace elements determination are still elaborated and required [23].

## 2. Results

### 2.1. Calibration Curve

In our experiments, a low resolution ICP-MS apparatus (described previously) was used to determine chlorine content based on ^35^Cl isotope signal, assuming that its interference with ^18^O^16^O^1^H should remain constant during the course of analysis and thus do not have any effect on determination results. Therefore, first the stability of this interferent signal for ultrapure water was determined. The observed intensities (c/s) for 10 measurements were relatively high (average: 27,986 ± 149), as they were coming either from ^18^O^16^O^1^H or from the trace of chloride present in ultrapure water, but very stable (relative standard deviation: RSD = 0.53%). It was then concluded, that such a stable analytical signal can be quantified as a constant in the calibration curve. Calibration curve prepared for chlorine showed linearity in whole investigated range (20–800 µg/L). This was proved by regression analysis as the correlation coefficient equaled 0.9997 (Table 1). A relatively low error of intercept (~2%) confirmed the stability of the interferent (^18^O^16^O^1^H) signal. Standard error of the regression allowed for the determination of limit of detection (LOD) as 15 µg/L and limit of quantification (LOQ) as 44 µg/L.

### 2.2. Determination of the Matrix Influence

As mentioned above analytical matrix itself may cause various interferences in ICP-MS measurements (called matrix effects), which can lead to a number of errors. Fortunately, not all the matrices elicit interferences in ICP-MS analysis. Therefore, an important step in our investigation was to determine the matrix influence on analytical signal of Cl during analysis of polyglycerols. Unfortunately, the compounds in question constitute multi-component mixtures of polyetherols with various molecular weights (MWs) (e.g., diglycerol, triglycerol, tetraglycerol) and there is a lack of commercially available single-compound standards to be used as matrices for evaluation of the analytical method. Moreover, there is no reference material of polyglycerol or similar compound with certified chlorine content. Since it was suspected that an increase in MW might increase the possibility for interference that affects determination results, polyethylene glycols (PEGs) with varying average MWs, as commercially available compounds of similar to polyglycerol structure, composition, and properties were used in place of polyglycerols. In ICP-MS it is recommended that total salt and organic matrix concentration in the sample should not exceed 1% (m/m), because of the hazard of clogging of the interfaces between ICP and MS [22]. To reduce the above risk, matrices of PEG concentration of 0.5% (m/m) and less were applied in our studies.

At first, it was observed that blank solutions of PEGs (solutions of PEGs without chloride) gave the same results as ultrapure water regardless of PEG type and concentration.

**Figure 1:**
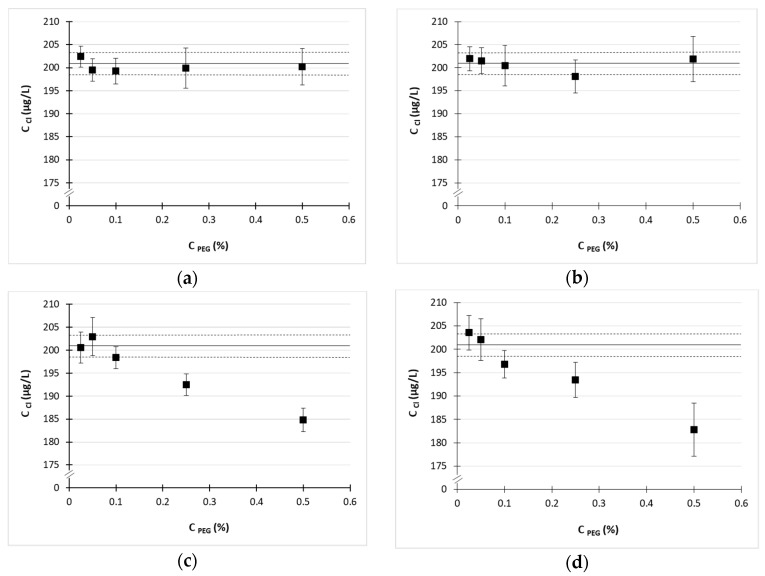
Results of the determination of chlorine in chloride spiked (200 µg/L) polyethylene glycols (PEGs) solutions: (**a**) PEG-600; (**b**) PEG-2000; (**c**) PEG-10000; (**d**) PEG-20000.

In Figure 1, the results of chlorine determination in chloride spiked (200 µg/L) PEGs solutions of various MWs and concentrations were provided. The horizontal solid line represents average result obtained for solutions without PEG (as a reference) while dotted lines border its standard deviation. In the case of PEGs of low average MW (<2000 Da) no significant influence of PEG content on chlorine determination results was observed. In contrast, matrices containing higher PEGs (10,000 and 20,000 Da average) gradually decreased the analytical signal of ^35^Cl at PEG concentrations higher than 0.1%, with a maximum decrease of approximately 10% for PEG-20000 at 0.5% (m/m).

Commercially available polyglycerols are generally of low MW, which lies in the range from 166 Da for diglycerol to 759 Da for decaglycerol. Therefore, their behavior should be similar rather to low MW PEGs, most likely PEG-600, than to heavier compounds. Thus, even a 0.5% solution of any polyglycerol should not elicit noticeable matrix effects during chlorine determination, as for PEGs with MW < 2000. This, when compared to previously determined LOQ for aqueous solution of chloride (44 µg/L), indicates the possibility of chlorine determination in polyglycerol samples even at level lower than 9 µg/g (LOQ of chlorine determination in polyglycerol). As mentioned in the introduction, since there are no specific regulations regarding chlorine content in polyglycerols applied in food and cosmetic industry, one may follow the EU (EC) in laying down specification for glycerol as a food additive, taking into account similar application of these two substances (polyglycerols and glycerol) in industry. The limit of chlorine content in food-grade glycerol equals 30 µg/g [4]. Therefore, our method is suitable for food-grade glycerol or polyglycerol quality control.

### 2.3. Effect of Inorganic and Organic Chlorine

Some authors reports that signal of chlorine in inductively coupled plasma atomic emission spectrometry (ICP-AES) and ICP-MS might depend upon the type of chlorine-containing compound itself [10]. Since in the proposed method chloride is used for calibration to determine the content of organically-bound chlorine, the above-mentioned discrepancy in analysis might hamper its results. To evaluate the extent of that, the effects of NaCl and α-monochlorohydrin (as a source of inorganic and organic chlorine respectively) on Cl determination were examined. Sodium chloride is the most popular component of analytical standards of chlorine (as chloride) and α-monochlorohydrin as glycerol derivate seems to be the best candidate for organic chlorine containing specie.

The results obtained indicated good accuracy and precision of the method studied, regardless of chlorine origin: they are consistent with the theoretical values (recovery close to 100%) and relative standard deviation does not exceed 2% (Table 2). In addition, statistical analysis (Student’s *t*-test, v = 4, α = 0.05) indicated that chlorine contents for inorganic and organic compounds were not statistically different (*t* = 1.15, *t*-critical = 2.776). It was then concluded that commercially available chlorine standards (containing NaCl) may be used for chlorine determination in PEGs and polyglycerols by direct ICP-MS despite the difference in the nature of chlorine bonding.

### 2.4. Determination of Chlorine in Polyglycerols

The method investigated was used to determine total chlorine in samples of polyglycerol, synthesized by the new method described elsewhere [24]. Epichlorohydrin constituted one of the main reagents for this synthesis, and the presence of both organic and inorganic chlorine in the product was very likely. Studied samples contained low MW polyetherols up to heptaglycerol and theirs average MW (ca. 500 Da) did not exceed studied range of MW of the lightest PEG-600 tested. Therefore, it was assumed that the whole investigated matrix concentration range could have been applied and 0.5% aqueous polyglycerol solutions were prepared. The results of determination of chlorine in these polyglycerol samples were shown in Table 3. The results obtained, despite a wide range of chlorine concentrations, were characterized by high precision (confidence interval up to 3% of measured value).

### 2.5. Validation of the Method

To validate the accuracy of chlorine determination by the method studied, Sample 1 was spiked with the amount of chloride standard sufficient to increase total chlorine content of 200 µg/L. Although the spiked Cl was not chemically bounded to the matrix, our previous study (Section 2.3) proved that Cl origin, bounded organic or free inorganic ion, does not influence the ^35^Cl signal during ICP-MS analysis. Additionally, according to the results provided in Section 2.2, the matrix of the sample (~0.1% polyglycerol content) also should not influence the analyte signal. Therefore, the result obtained for spiked sample should be the sum of Cl content coming from the sample and artificially added chloride. The results were shown in Table 4.

High recovery (99.7%) of the analyte with good precision (1.1%) was observed. This proved that the investigated method can be applied for direct determination of chlorine in polyglycerols.

Additionally, for complete validation of the method, Combustion Ion Chromatography System (CICS) was used as reference method. The results were shown in Table 5.

Statistical test (Student’s *t*-test, 5% significance level, three replicates) showed no significant difference between the results measured using our and reference methods. This proved the good accuracy of the presented ICP-MS method.

## 3. Materials and Methods

### 3.1. Reagents

Polyethylene glycol 600 (PEG-600), PEG-2000, PEG-10000, PEG-20000 and 3-chloro-1,2-propanediol (α-monochlorohydrin—analytical standard, Fluka, Buchs, Switzerland) supplied by Sigma-Aldrich (St. Louis, MO, USA) were used as model polyetherols and organically bound chlorine source, respectively. Standard solution of chloride (TraceCERT^®^ 1000 mg/L chloride in water (NaCl), Fluka) was used as calibration standard. Throughout the experimental work, ultrapure water (18 MΩ·cm, Simplicity Water Purification Systems, Milipore SAS, Molsheim, France) was used.

### 3.2. Apparatus

Chlorine content was determined with inductively coupled plasma mass spectrometer Varian 810-MS (Varian, Palo Alto, CA, USA). The operating parameters were as follows: RF power: 1.4 kW, plasma flow (argon): 17 L/min, auxiliary flow (argon): 1.7 L/min, nebulizer flow (argon): 1.00 L/min, pump rate: 4 rpm, sheath gas (argon): 0.2 L/min, nebulizer: micromist, spray chamber: Scott, quartz (3 °C), sampler cone: nickel; skimmer cone: nickel, number of scans: 10, *m*/*z*: 35.

As a reference method, Combustion Ion Chromatography System which consists of: (1) AQF-2100H Automatic Quick Furnace with GA-210 Gas Absorption Unit, produced by Mitsubishi Chemical Analytech (Enzo, Japan) and (2) Ion Chromatography System ICS-1100, Thermo Scientific (Waltham, MA, USA) was used. The conditions of the furnace were as follows: inlet temperature 1000 °C, outlet temperature 1100 °C, argon 200 mL/min, oxygen 400 mL/min. The conditions of ion chromatography were as follows: Dionex IonPacTM AS22 anion-exchange column (4 × 250 mm), temperature of column—30 °C, ASRS 500 suppressor and conductivity detector, eluent—4.5 mM Na_2_CO_3_/1.4 mM NaHCO_3_, flow—1.2 mL/min, calibration solutions were prepared using Thermo Scientific Seven Anion Standard II (Thermo Scientific, Waltham, MA, USA), the standard curve in the range of 0.1–10 mg/L.

### 3.3. Analytical methods

#### 3.3.1. Calibration curve

Calibration curve was prepared in the range of 20–800 µg/L using standard chloride solution. Linear regression analysis with Analysis Toolpack of Microsoft Excel (Microsoft Office Excel 2010, Microsoft, Redmond, WA, USA) allowed for the LOD and LOQ limits as respectively three and nine times standard error of the regression (“standard error” from Table 1) divided by slope of the standard curve (X Variable 1 from Table 1) [25].

#### 3.3.2. Determination of the Matrix Influence on Analysis Results

A 2.5% (*w*/*w*) solutions of PEGs of various MW (600, 2000, 10,000, 20,000 Da) were prepared by weighing an appropriate amount of PEG and dissolving with ultrapure water. Next, solutions containing 0%, 0.02%, 0.05%, 0.1%, 0.25%, 0.5% (*w*/*v*) of particular PEG and 200 µg/L chloride (from standard solution of Cl) were prepared. Blank solutions containing PEGs at the above concentrations were also prepared. The chlorine in the solutions was determined using ICP-MS, based on the previously described procedure.

#### 3.3.3. Determination of the Effect of Inorganic and Organic Chlorine

Solutions containing 0.1% matrix of PEG–2000 and 200 µg/L Cl in inorganic or organic form were prepared. Solutions with inorganic chlorine were obtained by the addition of chloride standard while solutions containing organically bound chlorine were prepared by the addition of appropriate amount of α-monochlorohydrin to the PEG–2000 solution. Six replicates were prepared in every case to enable statistical analysis of the results. The chlorine concentration in the solutions was measured using the ICP-MS method as described above.

#### 3.3.4. Determination of Chlorine in Polyglycerols

Solutions containing 0.5% of polyglycerols (synthesized using glycerol and epichlorohydrin applying various conditions) were prepared by dilution or dissolution of proper amounts of polyglycerol samples with ultrapure water. The conditions of the synthesis of these polyglycerols are not revealed because they are not relevant to these studies and are partially published elsewhere [24]. The chlorine concentrations in those solutions were measured using the above-mentioned ICP-MS method and calculated from the calibration curve. In the case of polyglycerols with chlorine content higher than 0.016 % (m/m) (160 µg/g, which after preparing 0.5% solution of polyglycerol gives the Cl concentration of 800 µg/L), solutions were diluted to match the chlorine content range in the calibration standard solutions (20–800 µg/L). All the measurements were performed at least in triplicate to enable statistical analysis of the results.

## 4. Conclusions

In our study, an ICP-MS method with basic equipment for direct chlorine determination in polyglycerol samples was developed. The range of linearity of calibration curve for ^35^Cl isotope was 20–800 µg/L with constant background signal coming probably from ^18^O^16^O^1^H interference. Limits of detection and quantification were determined to be 15 µg/L and 44 µg/L, respectively. The presence of water soluble organic species (matrix) similar to polyglycerols (polyethylene glycol—PEG) did not influence the chlorine determination results at matrix concentrations lower than 0.5% for PEGs with MW lower than 2000 Da and 0.1% for PEGs with MW lower than 20,000 Da. In addition, the results obtained indicated that chlorine can be determined with a good precision regardless of chlorine origin (inorganic/organic). Thus, it has been proven that the method studied can be successfully applied for determination of chlorine in polyglycerols with MWs lower than 2000 Da even at 9 µg/g level. This allows for quantification even under as restrictive requirements as EU regulation for chlorine content in a food-grade glycerol (less than 30 µg/g). Good accuracy and precision of the method was supported by high analyte recovery and low RSD values in the case of polyglycerol sample spiked with chloride as well as the result obtained by reference method.

## Figures and Tables

**Table 1:** Regression parameters of the calibration curve for ^35^Cl (R—correlation coefficient; df—the degrees of freedom in the source; SS—the sum of squares due to the source; MS—the mean sum of squares due to the source; F—the F-statistic)

**Regression Statistics**						
Multiple R	0.9999					
R Square	0.9997					
Adjusted R Square	0.9997					
Standard Error	1189					
Observations	9					
**ANOVA**						
	df	SS	MS	F	Signif. F	
Regression	1	39,431,146,027	3.94 × 10^10^	27901	7.27 × 10^−14^	
Residual	7	9,892,574	1,413,225			
Total	8	39,441,038,602				
	Coefficients	Stand. Error	*t* Stat.	*p*-value	Lower 95%	Upper 95%
Intercept	27,544	560	49.2	3.77 × 10^−10^	26,219	28,868
X Variable 1	241.9	1.45	167	7.27 × 10^−14^	239	245

**Table 2:** Accuracy and precision of ^35^Cl (originating from inorganic and organic compounds) determination (SD—standard deviation; RSD—relative standard deviation)

	Cl (µg/L)				
Chlorine Origin	Theoretical	Measured	SD	RSD (%)	Recovery (%)
NaCl	200	202.1	2.1	1	101.1
monochlorohydrin	200	200.3	1	0.5	100.1

**Table 3:** The results of chlorine determination in selected polyglycerol samples by using inductively coupled plasma mass spectrometry (ICP-MS) method developed in this work (average (x) ± confidence interval (u), *n* = 3, 95% confidence level).

Polyglycerol Sample	Cl (%)
	x ± u
1	0.033 ± 0.001
2	0.027 ± 0.001
3	0.0021 ± 0.0001
4	0.71 ± 0.02
5	0.37 ± 0.01
6	3.26 ± 0.09

**Table 4:** The results of determination of chlorine in sample no 1 spiked with chloride (200 µg/L). Matrix (PGL no 1) concentration ~0.1%, *n* = 4.

	Cl (µg/L)				
Sample	Sample	Spiked Sample	Theoretical	Recovery (%)	RSD (%)
1	357.1	555.2	557.1	99.7	1.1

**Table 5:** The results of determination of chlorine in sample 2 by elaborated (ICP-MS) and reference (CICS) methods. Recovery concerns ICP-MS method relative to the reference one (CICS).

	Cl (µg/g)		
Sample	ICP-MS	CICS	Recovery (%)
2	273 ± 4	272 ± 8	100.4
